# Supplementary material for: Activin A Limits VEGF-Induced Permeability via VE-PTP
Source: Int J Mol Sci. 2023 May 12;24(10):8698. doi: 10.3390/ijms24108698 (PMC10218593; doi:10.3390/ijms24108698)
Supplement: Supplementary file 1 [file ijms-24-08698-s001.zip › ijms-2381018-supplementary.pdf]

Supplementary figures

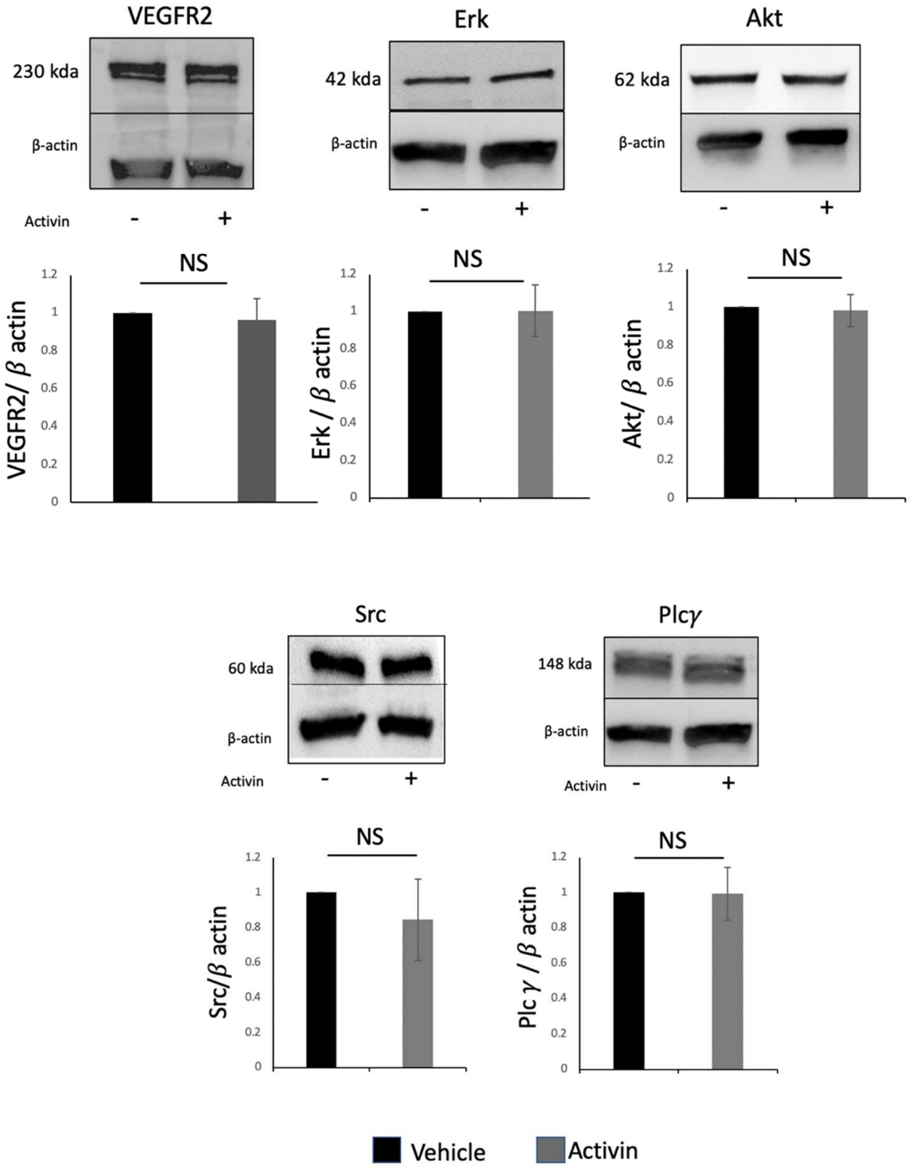

Figure S1: Activin had no effect on most reduces permeability without interfering with VEGF effectors.
Cells were treated with vehicle or activin for 48h, lysed and the clarified lysates were subjected to western blot analysis using the indicated antibodies. The bar graphs show the total protein level of VEGFR2, Src, Plcγ, Erk and Akt normalized to the level of βactin in 3 independent experiments. Uncropped full-length blots are presented in Figure S8.

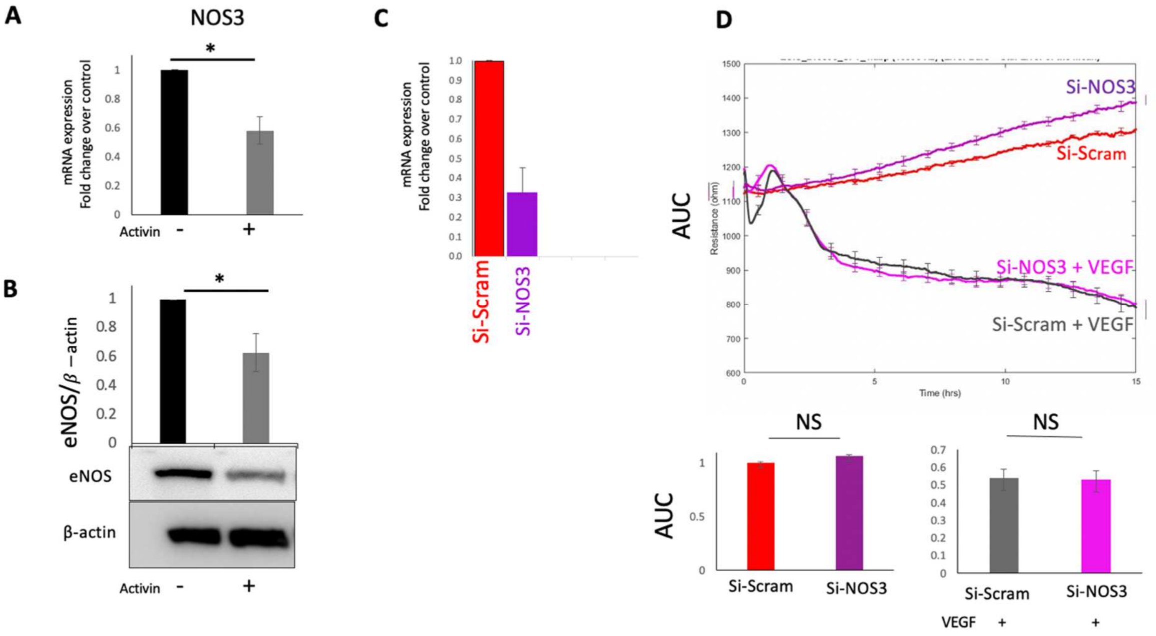

Figure S2: Suppressing eNOS expression did no attenuate VEGF-induced permeability.
A) Confluent HRECs were treated for 48h with either vehicle (black bar) or activin (gray bar), harvested and subjected to qRT-PCR analysis. The data in the bar graph are the mean +/-SEM change in expression in response to activin (n= 3-4). At least 3 independent experiments showed similar results. *P< 0.05.
B) Cells were treated as described in A, lysed and subjected to Western blot analysis using the indicated antibodies. The images are of representative Western blots; the bar graphs show the mean +/- SEM change in expression in response to activin in 3 independent experiments. *P < 0.05. Uncropped full-length blots are presented in Figure S9.
C) HRECs were transfected with untargeted (si-Scram) or NOS3-targeted (si-NOS3) siRNA and after 48 h lysed and subjected to qRT-PCR analysis as in panel A.
D) Cells were transfected with the indicated siRNAs and then stimulated with PBS or 2 nM VEGF. Permeability was monitored as described in Fig 1. The data (area under the curve (AUC) from 0-15 h)) was quantified and is presented in the bar graphs; the effect of the two siRNAs on cells stimulated with PBS and VEGF is on the left and right, respectively. Similar results were observed in 3 independent experiments.

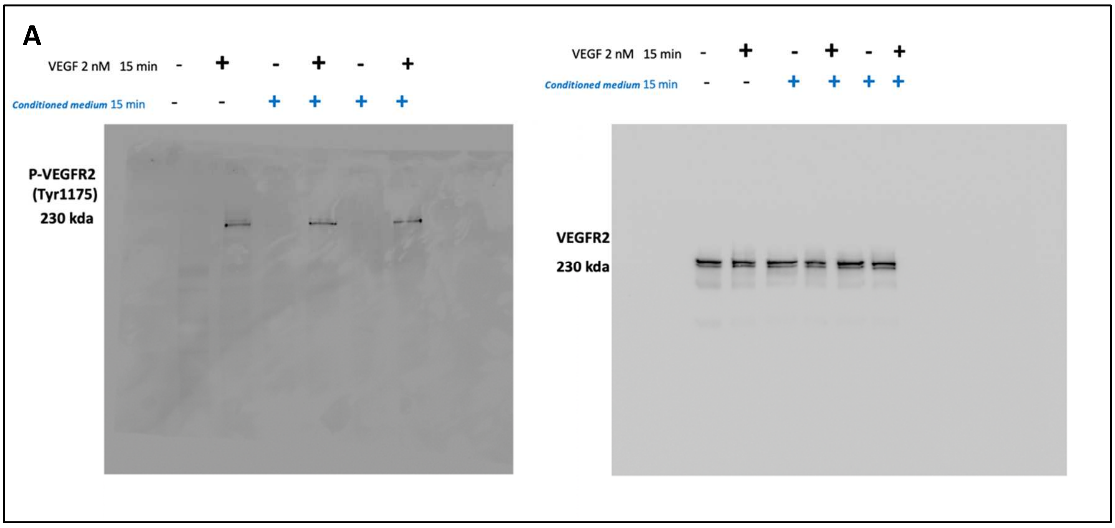

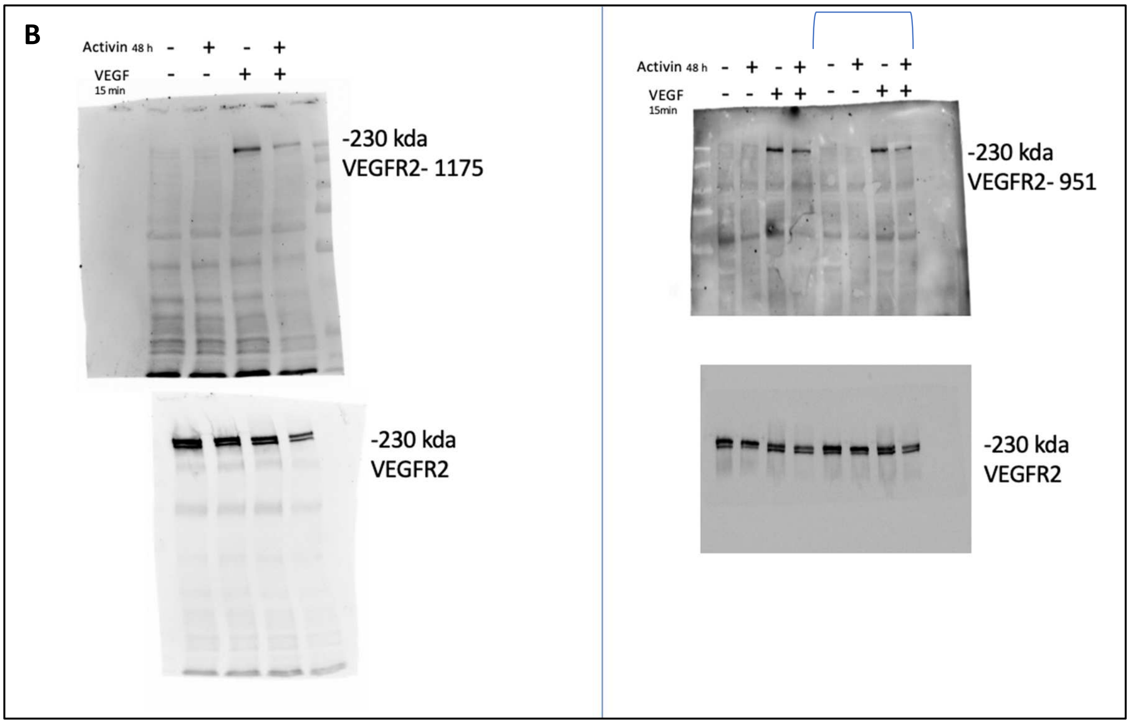

Figure S3: Uncropped Western Blots images of Fig 3. Representative Western blot images with molecular weight standard show the relative change of VEGFR phosphorylation
A) In conditioned medium experiment. Stripped membranes from P-VEGFR1175 (left) were reblotted for VEGFR2 (right).
B) Following 48h activin pretreatment, samples derive from the same experiment and were processed on the same gel. Stripped membranes from A) P-VEGFR 1175 and B) P-VEGFR 951 were reblotted for VEGFR2.

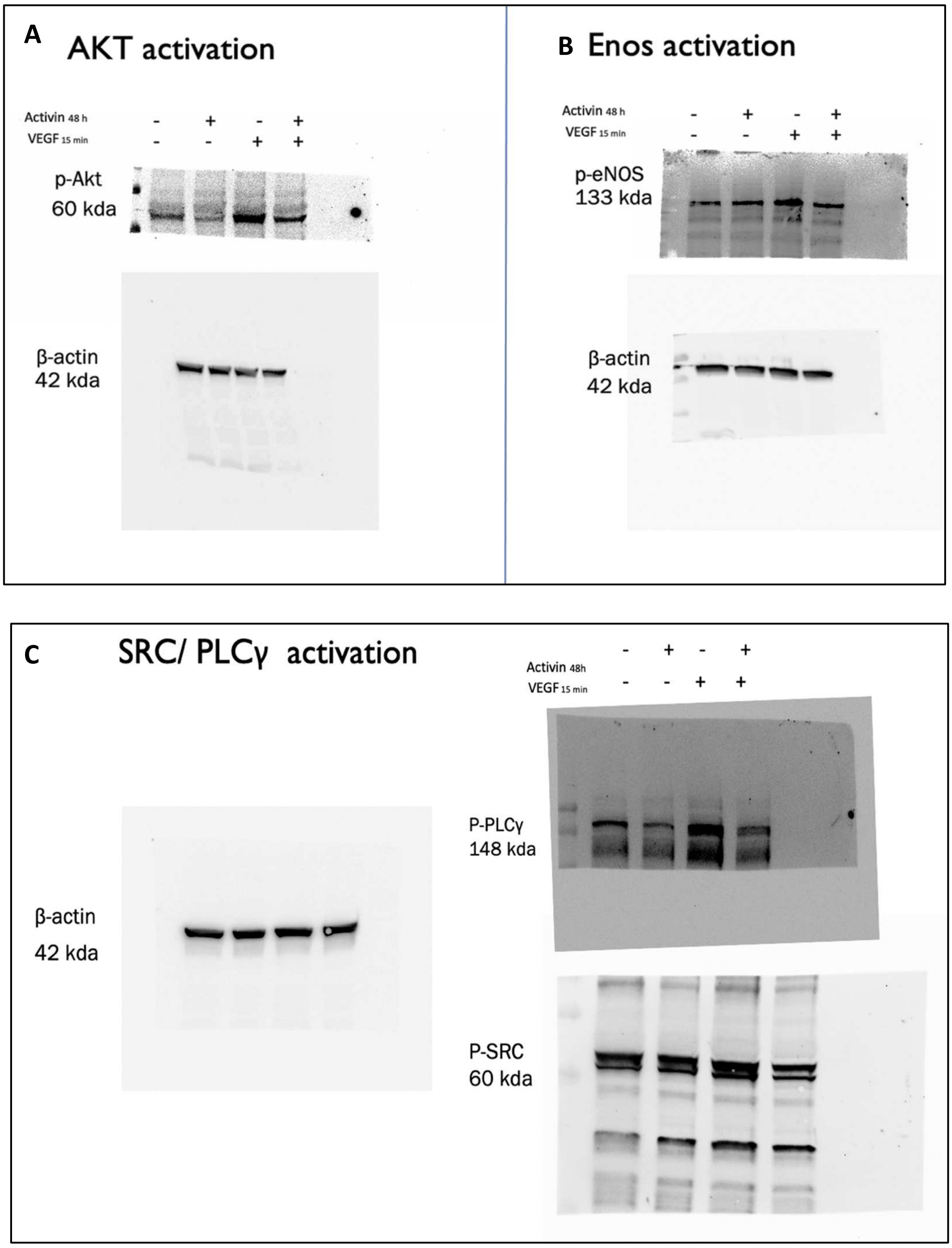
A
AKT activation
Activin 48 h
VEGF 15 min
p-Akt
60 kda
β-actin
42 kda
B
Enos activation
Activin 48 h
VEGF 15 min
p-eNOS
133 kda
β-actin
42 kda
C
SRC/ PLCγ activation
β-actin
42 kda
Activin 48h
VEGF 15 min
P-PLCγ
148 kda
P-SRC
60 kda

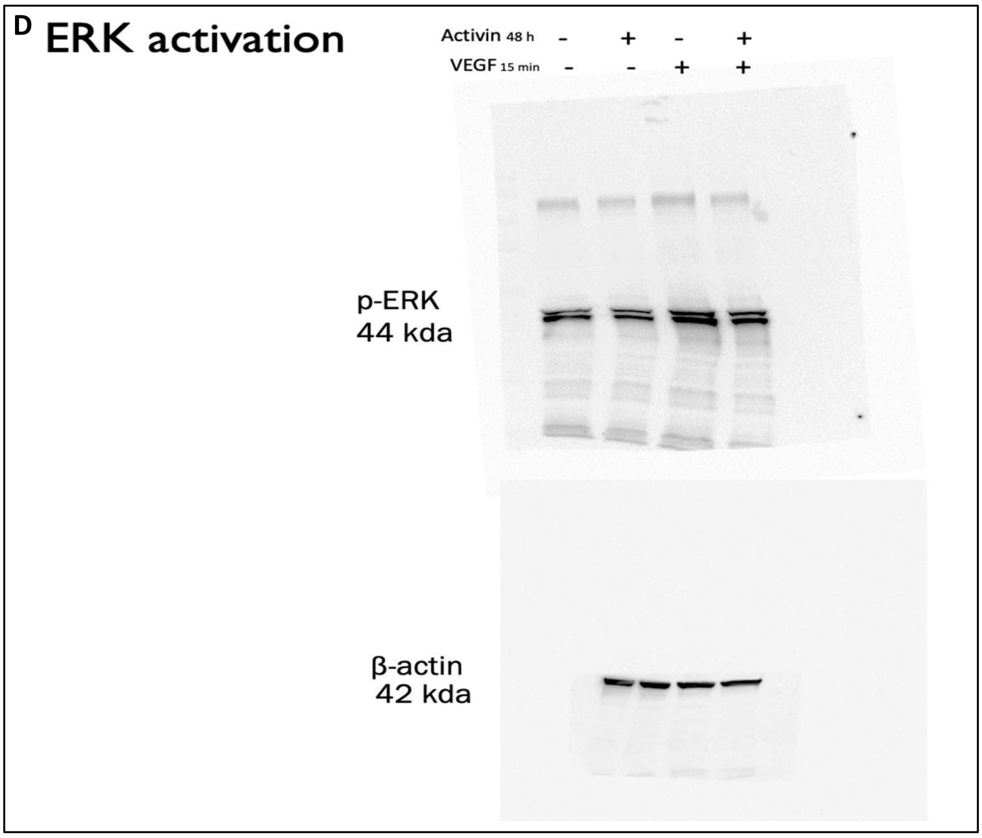

Figure S4: Uncropped Western Blots images of Fig 4.
The images are of representative Western blots with molecular weight standard showing the relative change in the extent of activation of several VEGF downstream effectors in activin-treated cells. Samples deriving from the same experiment were processed on the same gel. In A, B, C, and D, blots were cut based on the molecular weight of the targeted proteins.

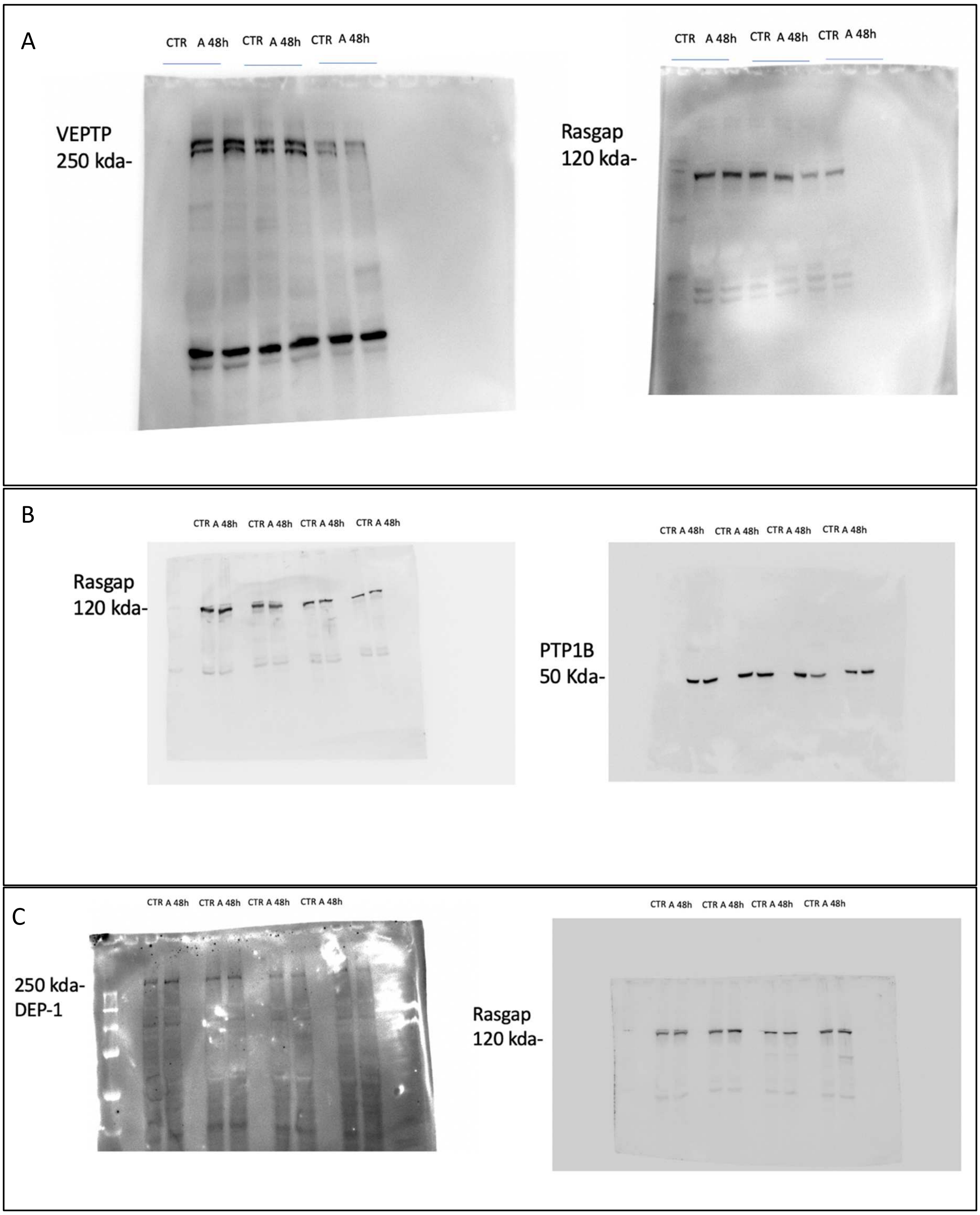

Figure s5*:* Uncropped Western Blots images of Fig 5.
Representative Western blots images with molecular weight standard show the relative change of PTPs levels in 48h activin treated cells. Stripped membranes from A) VEPTP, B) PTP1B and C) DEP-1 were reblotted for RasGAP.

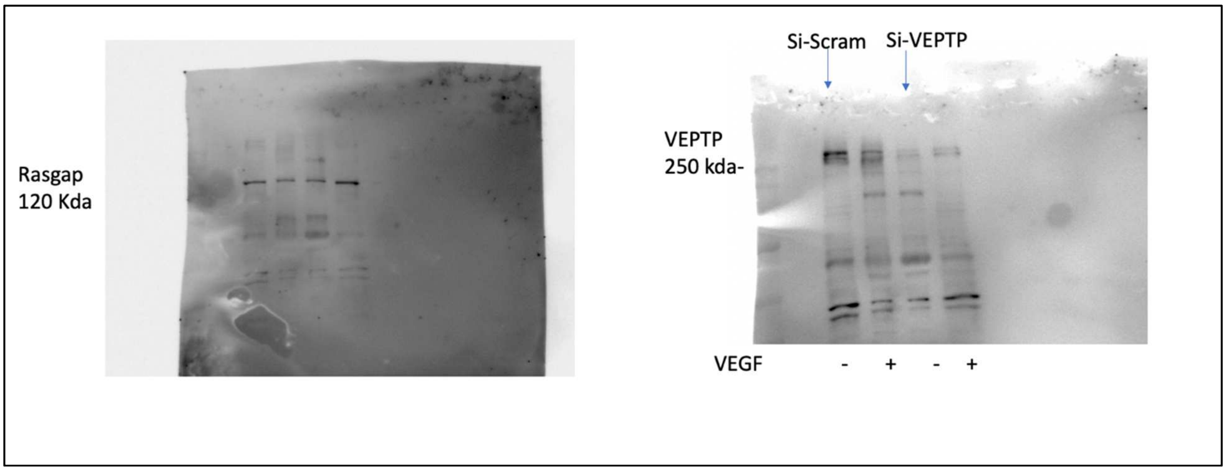

Figure s6: Evaluation of VEPTP level following deletion of PTPRB expression. HRECs were transfected with untargeted (si-Scram) or PTPRB-targeted (si-PTPRB) siRNA and after 72 h lysed and subjected to Western blot analysis with the indicated antibodies. Uncropped representative Western blots image with molecular weight standard show the extent of decrease of VEPTP level. Stripped membranes from VEPTP were reblotted for RasGAP.

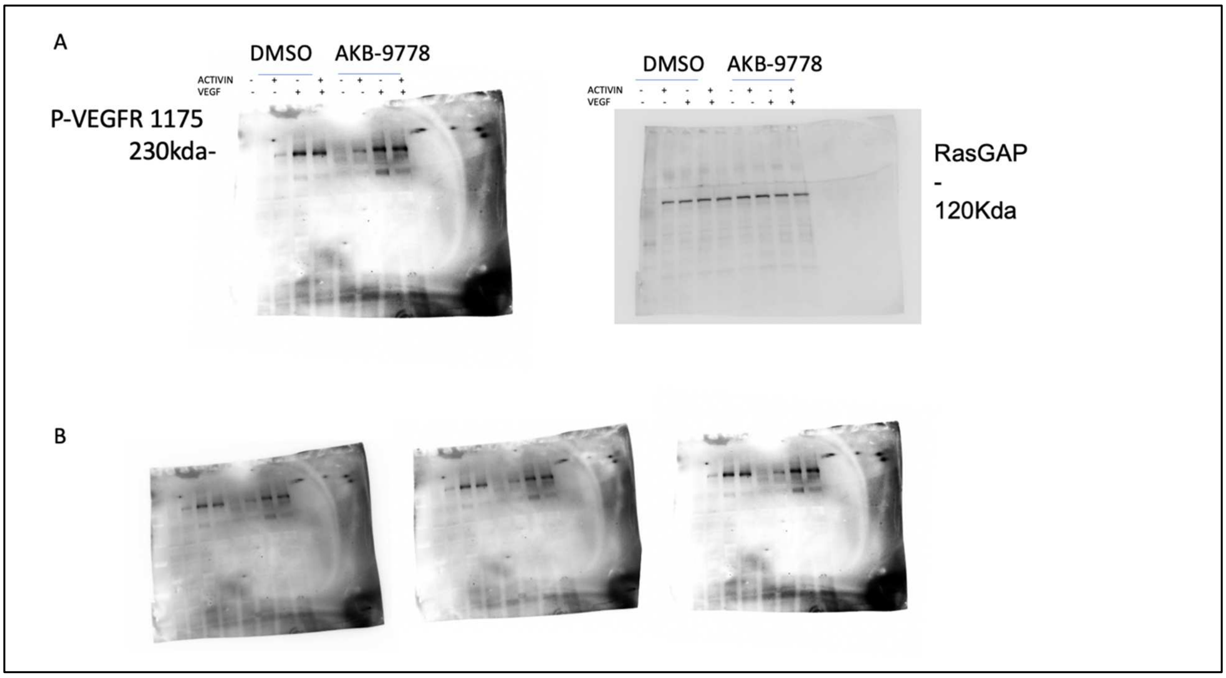

Figure s7: Uncropped Western Blots images of Fig 7.
A) Representative Western blots images with molecular weight standard showing the relative change of VEGFR2 phosphorylation following pretreatment with VEPTP inhibitor. Stripped membranes from P-VEGFR2 1175 were reblotted for RasGAP. B) Blot images of multiple exposures of Western blots images of P-VEGFR2 1175.

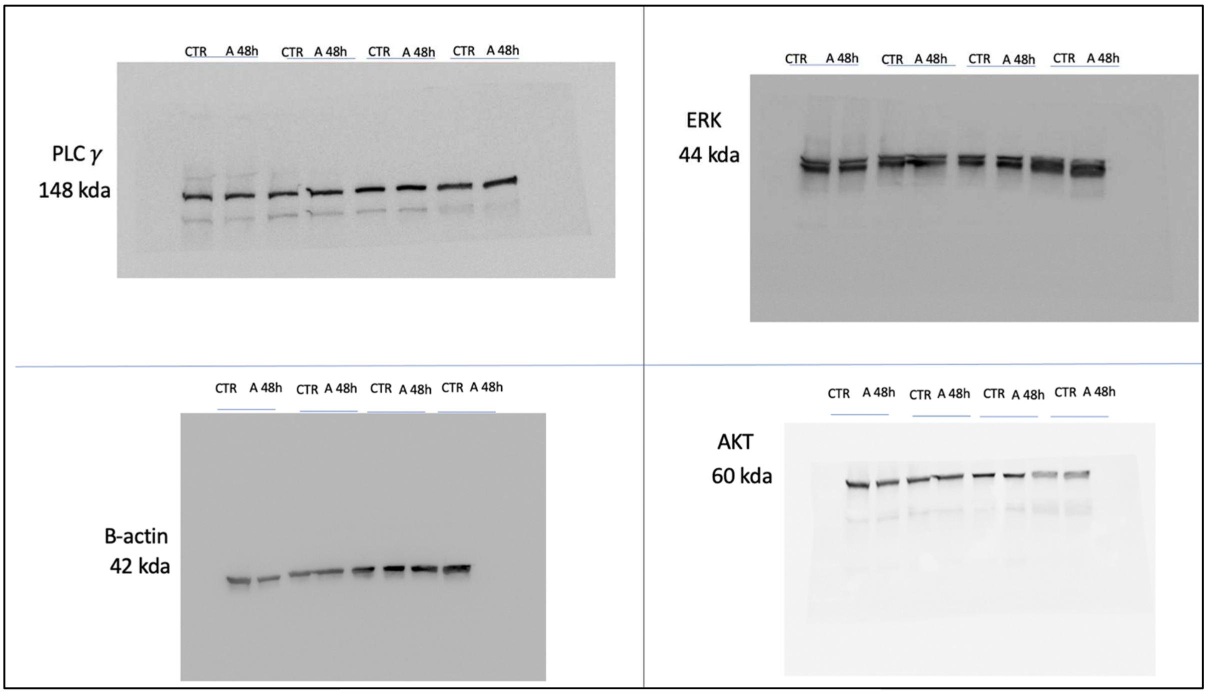
CTR A 48h CTR A 48h CTR A 48h CTR A 48h
PLC γ
148 kda
CTR A 48h CTR A 48h CTR A 48h CTR A 48h
ERK
44 kda
CTR A 48h CTR A 48h CTR A 48h CTR A 48h
B-actin
42 kda
CTR A 48h CTR A 48h CTR A 48h CTR A 48h
AKT
60 kda

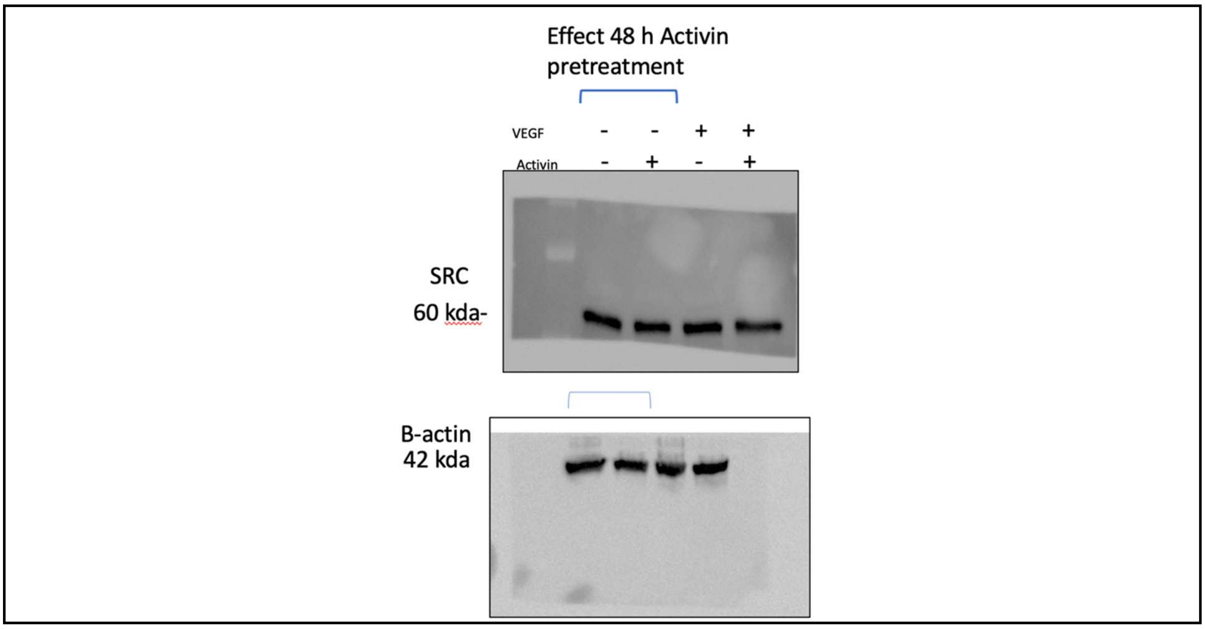
Effect 48 h Activin
pretreatment
VEGF - - + +
Activin - + - +
SRC
60 kda-
B-actin
42 kda

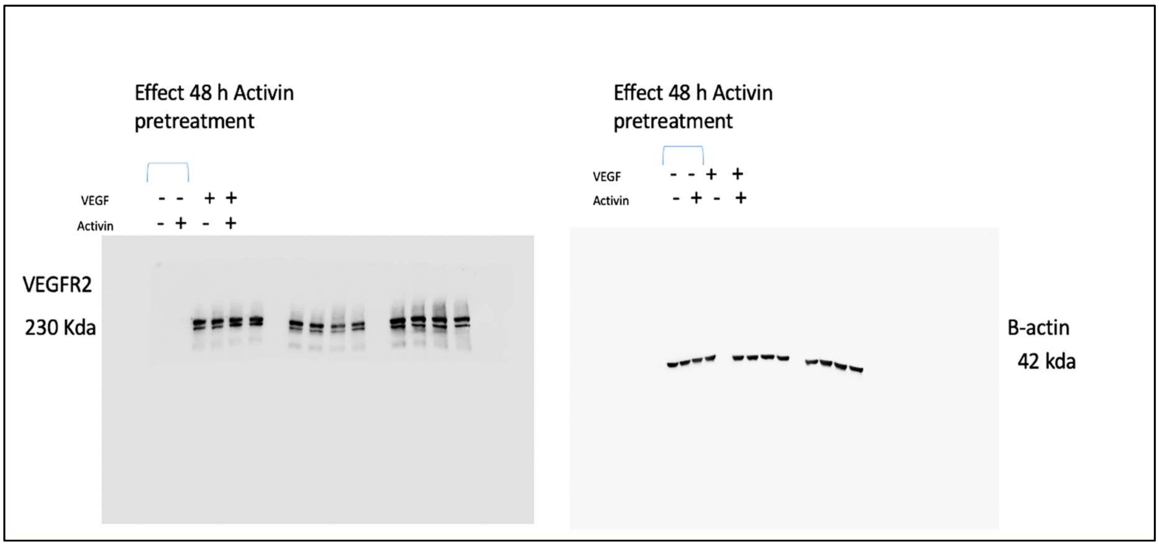

Figure s8: Uncropped Western Blots images of Figure s1. The images are of representative Western blots with molecular weight standard, showing the effect of activin on several VEGF effectors. Stripped membranes from Erk were reblotted for Akt. Blots were cut based on the molecular weight of the targeted proteins.

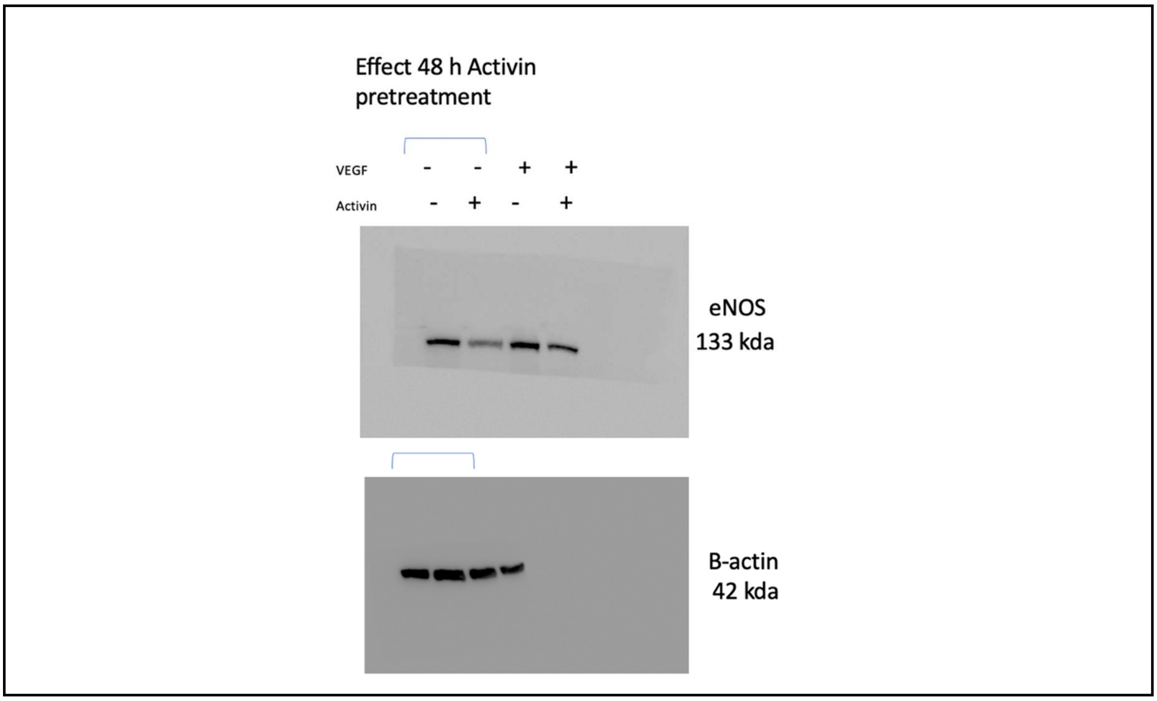

Figure s9: Uncropped Western Blots images of Figure s2. The images are of representative Western blots with molecular weight standard showing change of eNOS expression in activin treated cells. Blots were cut based on the molecular weight of the targeted proteins.

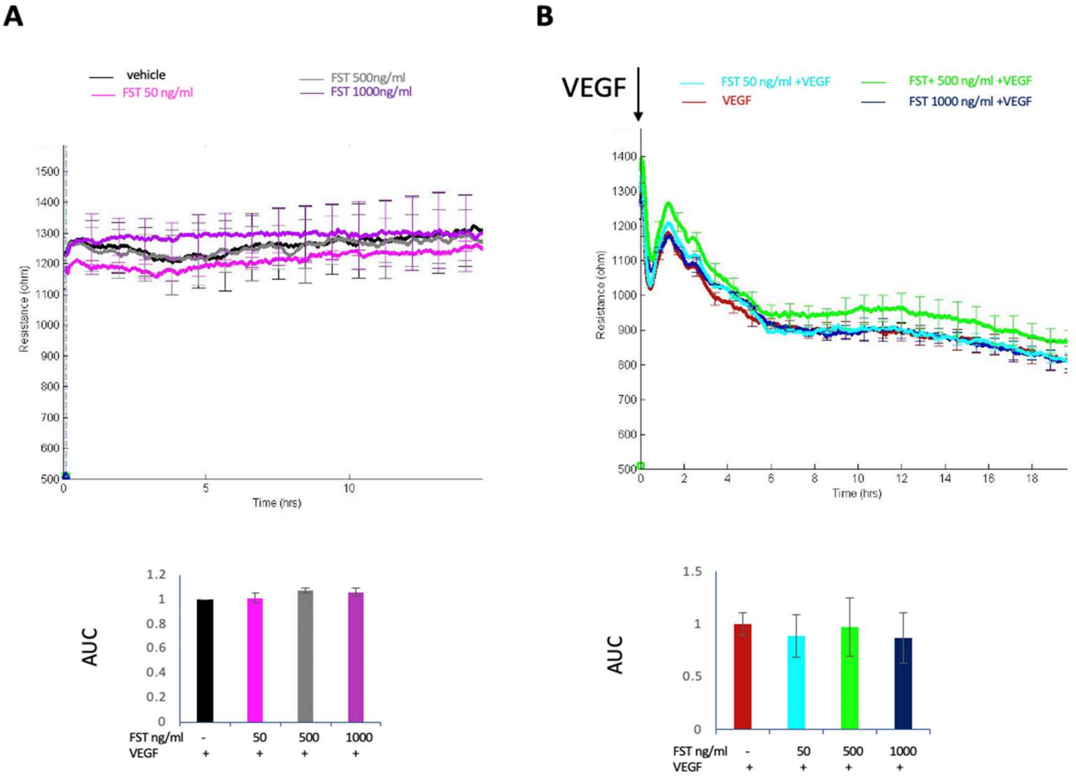

Figure s10: Recombinant FST had no effect on basal and VEGF-stimulated barrier.

Permeability of HRECs was continuously recorded in:

(A) Cells treated with FST vehicle (black), FST 50 ng/ml (pink), 500 ng/ml (grey) and 1000 ng/ml (purple).

(B) Cells stimulated with 2 nM VEGF alone or VEGF in combination with FST 50 ng/ml (cyan), FST 500 ng/ml (green), or FST 1000 ng/ml (dark blue).

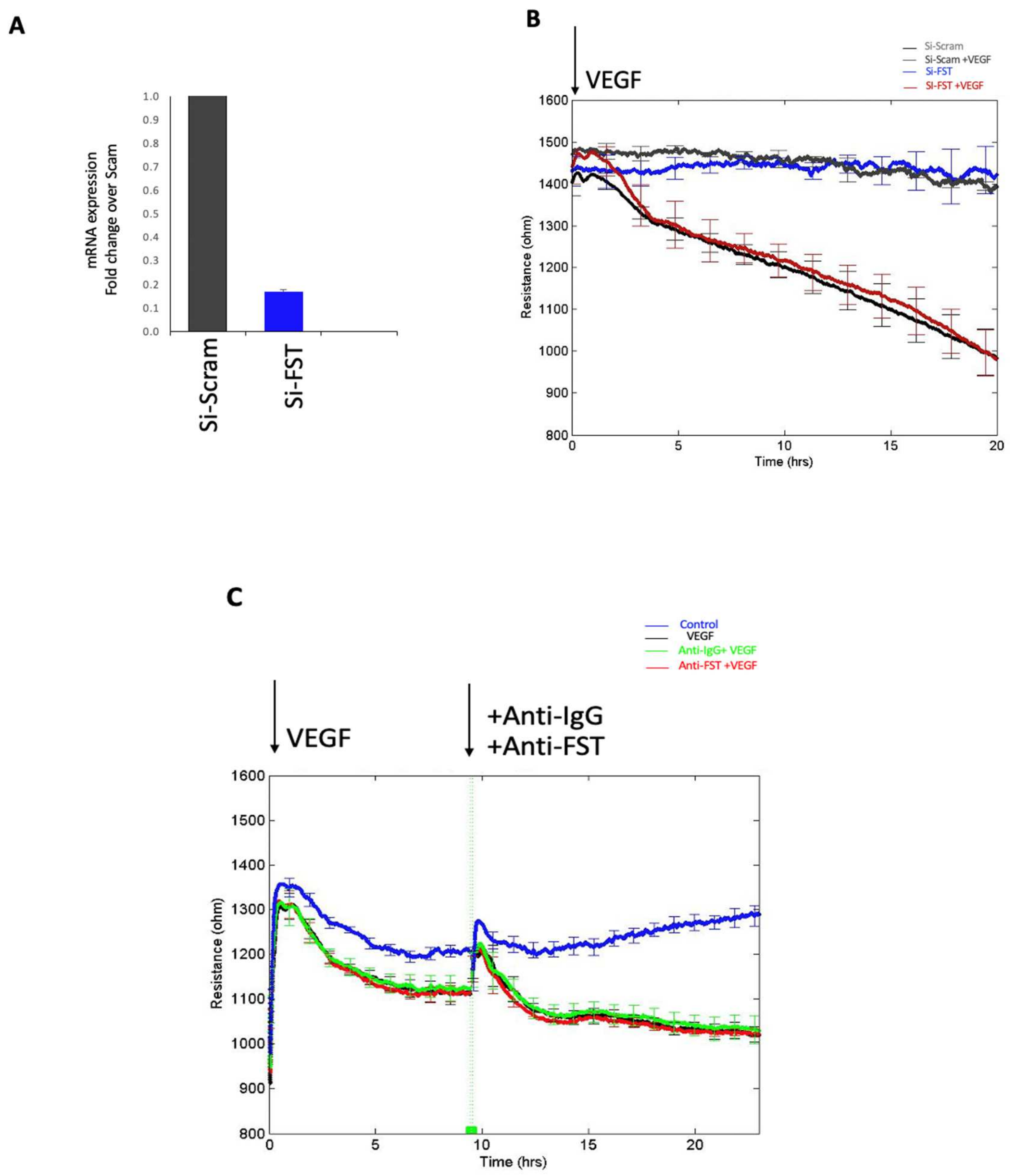

Figure s11: Neutralizing FST had no effect on VEGF-mediated barrier opening and re-closure.

(A) HRECs were transfected with untargeted (si-Scram) or FST-targeted (si-FST) siRNA, lysed and subjected to qRT-PCR. The data in the bar graph represents change in FST expression relative to untargeted cells. (B) HRECs that had been transfected with the indicated siRNAs, for 48 h were stimulated with PBS or 2 nM VEGF, and permeability was monitored as described in Fig 1. (C) The permeability of HRECs that were first exposed to VEGF for 10 h followed by addition of anti-IgG (green), anti-FST (blue) or anti-FST vehicle (dark red) was monitored as described in Fig 1. Arrows indicate when VEGF, anti-IgG and anti-FST were added.
